# Unusual Urologic Metastasis From Gastrointestinal Cancers: A Compilation of Case Reports and Literature Review

**DOI:** 10.7759/cureus.33647

**Published:** 2023-01-11

**Authors:** Ana Barbosa, Vânia Grenha, Ilda Faustino, Camila Coutinho

**Affiliations:** 1 Oncology, Hospital Senhora da Oliveira, Guimarães, PRT; 2 Urology, Hospital Senhora da Oliveira, Guimarães, PRT

**Keywords:** clinical case, haematuria, penile metastasis, bladder metastasis, gastrointestinal cancer

## Abstract

Gastrointestinal cancers are highly prevalent around the world. In the metastatic setting, the most usual sites for metastases are the liver, lymph nodes, peritoneum, and lung. Urologic metastases are very rare.

We report a case series of three patients with gastrointestinal tumours in different topographies (stomach, colon, and rectum) with urological metastases. In all cases, the patients were initially treated with curative intent. Two of the patients presented with bladder metastases, and the third had penile metastases in addition to pulmonary metastases. Haematuria was the most common symptom at presentation. One of the patients had a good overall survival and is still undergoing palliative intent chemotherapy.

In the literature, there are few reported cases of urological metastases from gastrointestinal cancers, and that is the aim of this publication.

## Introduction

Gastrointestinal tumors account for 26% of the global cancer incidence burden and 35% of all cancer-related deaths. In stage IV gastrointestinal tumors, the most common sites for metastasis are the liver, lymph nodes, peritoneum, and lung [1]. Bladder metastases are rare and represent less than 2% of bladder neoplasms [2]. The most common sites of origin of secondary bladder neoplasms include the colon, prostate, endometrium, cervix, breast, and lung [3]. Penile metastases are rare, and the most common sites of origin are the prostate and bladder [4,5]. The pathophysiology of bladder and penile metastasis from the gastrointestinal tract is still poorly understood as it is a sporadic event [6].

Our article aims to present a mini-series of three cases of gastrointestinal cancers with urological metastases and their poorly understood physiology.

## Case presentation

Clinical case 1

**Figure 1:**
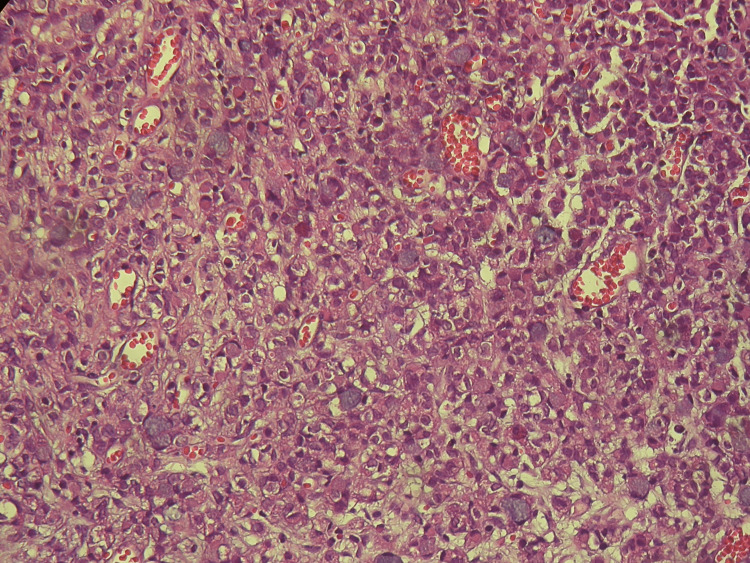
Signet ring cell carcinoma infiltrating the bladder Microscopic appearance using haematoxylin and eosin stain; original magnification: 100x

A 53-year-old female, with Eastern Cooperative Oncology Group Performance Status (ECOG PS) 0, and a background history of hypertension and dyslipidemia. Due to heartburn, nausea and postprandial fullness, she underwent an upper digestive endoscopy with a biopsy, which revealed a gastric adenocarcinoma. Complete staging (including diagnosis laparoscopy) was cT4N+M0. The case was discussed in a multidisciplinary team meeting, and the patient was proposed for perioperative chemotherapy (ChT) with cisplatin/5-fluorouracil (CF) followed by gastrectomy. The anatomopathological examination of the surgical specimen revealed “slightly cohesive cells with infiltrating signet ring type,” staged as ypT3N2G3. She completed three cycles of CF after surgery in June 2017. A year after the completion of ChT, the patient started complaining of incontinence and haematuria. She underwent a bladder ultrasound, which revealed “bladder thickening and left pyelocaliceal dilatation; a transurethral resection of a 4cm lesion of the bladder fundus revealed an adenocarcinoma with signet ring cell morphology compatible with a bladder metastasis of the gastric neoplasia” (Figure 1). Palliative ChT with 5-fluorouracil/oxaliplatin (FOLFOX6) regimen was started; however, the patient died after the third cycle due to disease progression.

Clinical case 2

**Figure 2:**
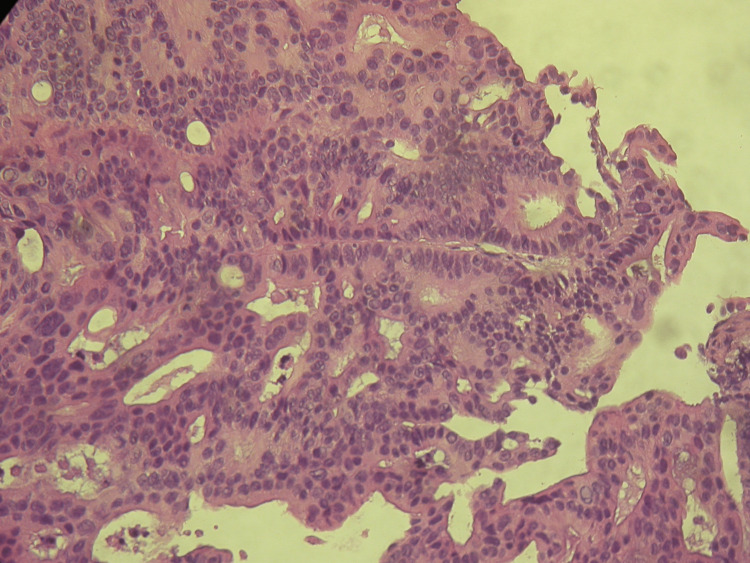
Malignant epithelial neoplastic structures of adenocarcinomas invading the bladder Microscopic appearance using haematoxylin and eosin stain; original magnification: 100x

**Figure 3:**
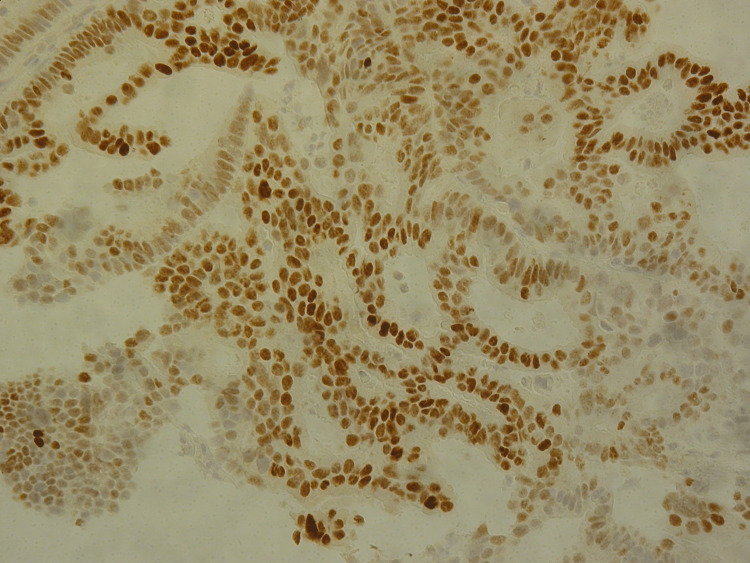
Immunohistochemistry revealing Caudal-type homeobox transcription factor 2 (CDX2) positive tumour cells Original magnification: 100x

An 84-year-old female, with ECOG PS 1, without relevant medical history. The patient was hospitalised due to intestinal obstruction and was diagnosed with a stenosing neoplasm of the hepatic flexure of the colon. She underwent an exploratory laparotomy with a right hemicolectomy. The anatomopathological study revealed a low-grade invasive adenocarcinoma (G1-well-differentiated), staged as pT4aN1cM0. As decided in a multidisciplinary team meeting, the patient was proposed for adjuvant ChT with capecitabine, at a dose adjusted for the patient’s age and performance. She completed eight cycles of adjuvant ChT with good tolerance. A year after completion of ChT, a CT scan of the chest, abdomen and pelvis was performed, which revealed right hydronephrosis and a nodular lesion in the transition between the ureters and the iliac vessels, which were not approachable for biopsy. Given the worsening of renal function, one month later, a new abdominopelvic CT scan was performed, which revealed an increase in the tumour lesion on the right lateral wall of the bladder, with the major axial axes measuring 35mm x 32mm. Biopsy of the bladder lesion revealed tissue fragments with malignant epithelial neoplasm, and an immunohistochemical profile compatible with bladder metastasis of colonic origin (Figures 2, 3): caudal-type homeobox transcription factor 2 (CDX2) +, cytokeratin (CK) 7-, CK20-, GATA binding protein 3 (GATA3), and thyroid transcription factor (TTF-1). The patient’s general condition worsened quickly, and the patient died six months after the relapse without being able to receive palliative ChT. The patient received supportive care during these months.

Clinical case 3

**Figure 4:**
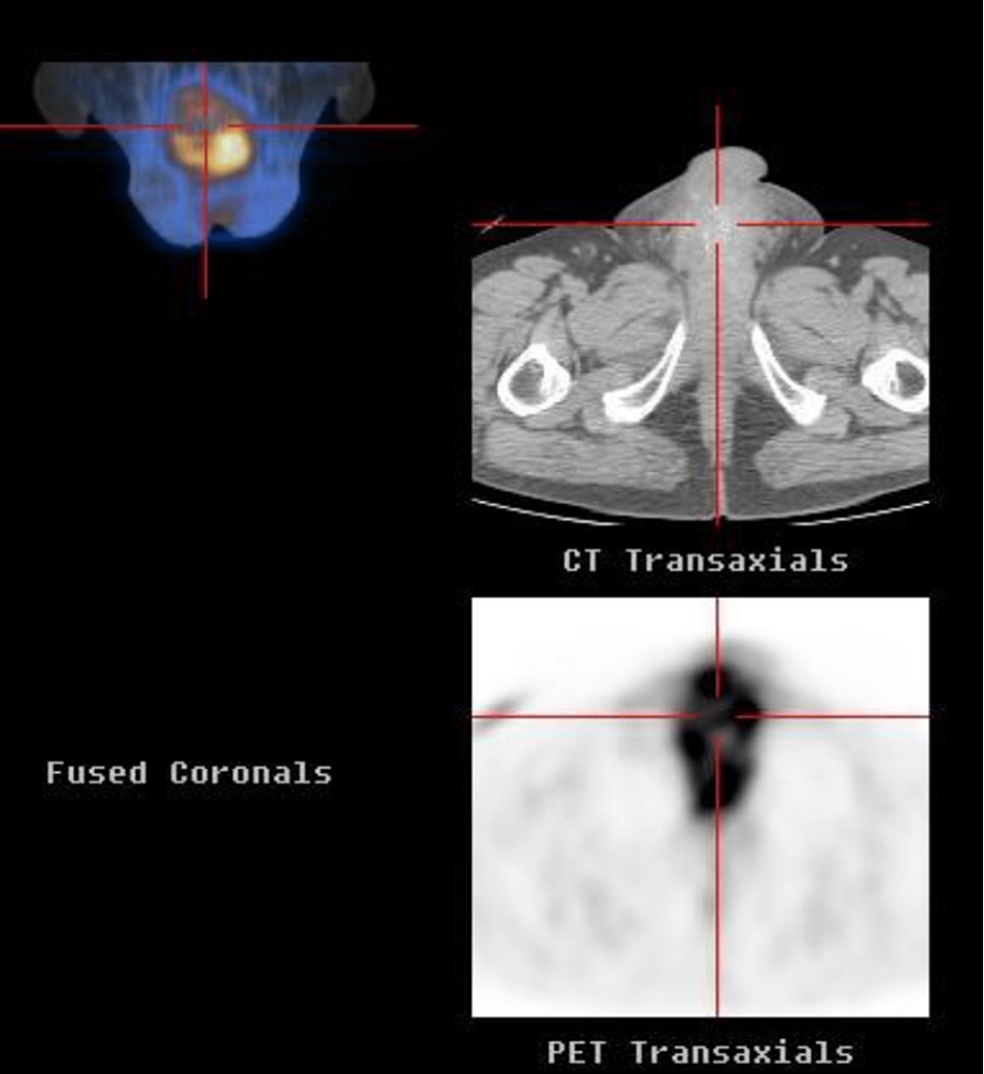
18F-FDG PET showing penile metastasis FDG PET: Fluorodeoxyglucose positron emission tomography

**Figure 5:**
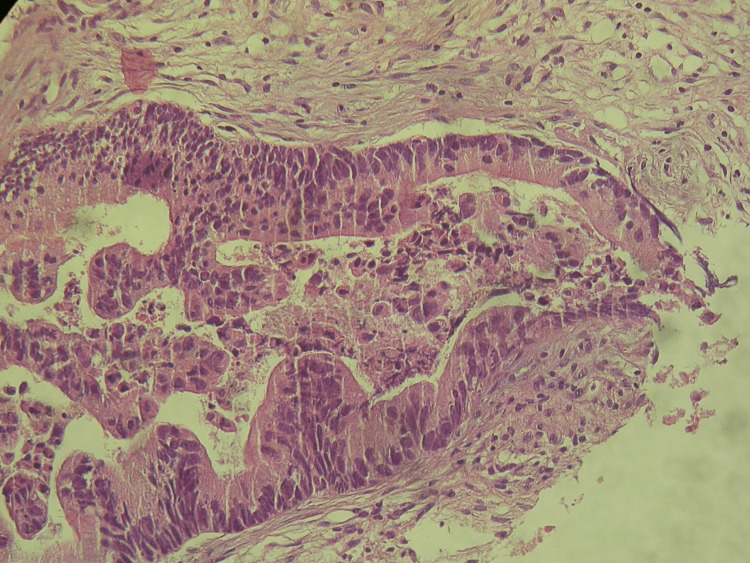
Malignant epithelial neoplastic structures of adenocarcinoma invading the penile structures Microscopic appearance using haematoxylin and eosin stain; original magnification: 100x

**Figure 6:**
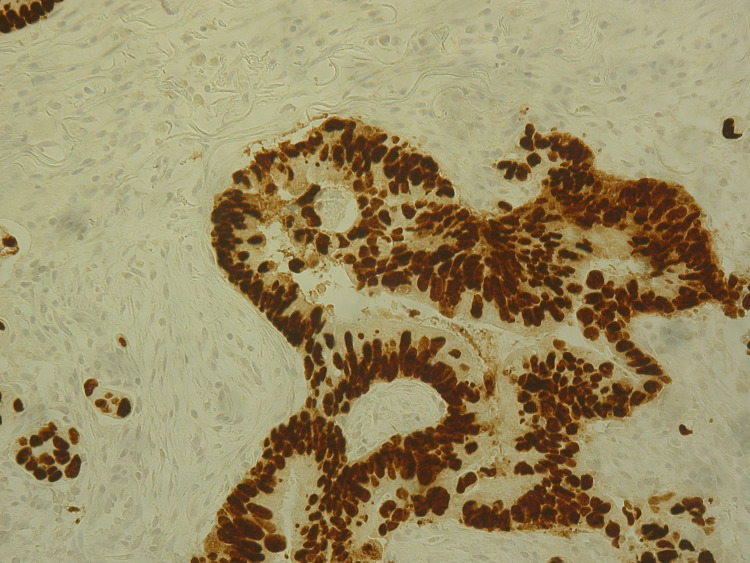
Immunohistochemistry cytokeratin 20 (CK20) positive tumour cells Original magnification: 100x

A 56-year-old male, with ECOG PS 0, former smoker without relevant medical history. In 2012, he was diagnosed with an adenocarcinoma of the rectum, cT3N+M0. In this context, he was offered concomitant ChT with 5-fluorouracil (5-FU) and radiotherapy (RT), followed by a complete anterior resection of the rectum, ypT3N0G1. After surgery, he completed 12 cycles of adjuvant ChT with FOLFOX6. Six years later, he started complaining of haematuria and a progressively growing ulcer with a hard consistency at the base of the penis. In this context, he performed an abdominopelvic nuclear magnetic resonance (AP NMR) and an 18- fluorodeoxyglucose positron emission tomography (PET), which revealed an extensive penile mass causing obliteration of the penile urethra (Figure 4). A biopsy was performed on the penile lesion, and its histology revealed malignant epithelial neoplastic structures of adenocarcinoma with an immunohistochemical profile of CK20+, CDX2+ and CK7-, compatible with metastatic neoplasia from primitive adenocarcinomas of the colon/rectum (Figures 5, 6). The molecular profiles of neuroblastoma RAS viral oncogene homolog (NRAS), Ki-ras2 Kirsten rat sarcoma viral oncogene homolog (KRAS), and proto-oncogene B-Raf and v-Raf murine sarcoma (BRAF) were wild-type. There was no neurotrophic tyrosine receptor kinase (NTRK) rearrangements or microsatellite instabilities. On the chest CT scan, pulmonary metastases were also observed. The patient started palliative ChT with 5-FU/irinotecan (FOLFIRI) and bevacizumab in January 2019. A year later, due to pulmonary disease progression, he started second-line ChT with FOLFOX6 and bevacizumab. In November 2021, he showed clinical and imagiological progression of the penile lesion, with stability in the lung disease. Given local progression only in the penile lesion, it was decided to perform local RT at a dose of 30 Gy in 10 fractions and restart FOLFOX6. In February 2022, the patient presented with lung disease progression and started third-line ChT with trifluridine/tipiracil (TAS-102).

## Discussion

This compilation of cases show the history of three patients with gastrointestinal tumours in three different topographies: stomach, colon, and rectum. All were initially treated with curative intent, and, interestingly, all presented haematuria at the time of relapse.

In clinical case 1, it’s important to note that the 'signet ring type' is an aggressive histology with a poor prognosis that frequently presents with different metastatic patterns (the peritoneum and ovary being the most common sites) [3]. The pathophysiology of metastasis to the bladder is unclear, but transcoelomic migration seems to be the most likely [2]. Mitsimponas (&) Zervopoulos reviewed case reports in the pertinent international medical literature and found 24 case reports involving 28 patients with secondary bladder tumours from primary gastric neoplasms [7].

In clinical case 2, the patient presented with an adenocarcinoma of the colon, stage III, with high-risk factors (pT4 and an occlusive condition at diagnosis), which may confer a worse prognosis. In this case, the immunochemistry with CK7-, CK20+, and CDX2+, is a frequently seen pattern in gastrointestinal cancers. As a result, immunohistochemistry was decisive for differentiating primary from metastatic adenocarcinoma. Despite not having any relevant previous medical history, the patient’s advanced age at diagnosis may also have played an important role in the evolution of this case. Older age may confer frailty due to a decreased physiologic reserve, resulting in a worse tolerance to aggressive treatments and possibly a more rapid performance degradation when the disease relapses.

In clinical case 3, we presented a case of a young man treated with a multimodal radical treatment for locally advanced rectal cancer. He presented with metastatic disease six years after curative intent treatment. A PET or NMR are adequate non-invasive methods for evaluating the extension of lesions, but a biopsy is mandatory for the confirmation of the diagnosis of metastasis. Treatment is palliative in the majority of cases and can include surgical excision, RT, ChT, and symptomatic management. In our patient, the decision for ChT was based on the presence of pulmonary metastasis. However, when he presented only local penile progression, he was submitted to local treatment with RT. Metastasis to the penis typically indicates advanced-stage disease, and the prognosis is poor, with half of the patients dying within one year of diagnosis [8,9]. This metastasis location is probably related to the retrograde venous route, followed by lymphatic and direct invasion [8-10]. Kuliavas et al. refer to secondary malignancy of the penis as an uncommon clinical entity with less than 40 cases reported [10]. Our case is unusual not only because the penile lesion was the initial presentation of disease relapse but also because this patient has been receiving palliative treatment for nearly four years with good tolerance and maintaining an ECOG PS 1.

## Conclusions

This article describes three different clinical cases in which metastases of gastrointestinal tumours occurred in urologic sites. The authors presented these clinical cases due to their rarity.

All patients were initially treated with curative intent, but all had high-risk factors for recurrence. Metastasis topographies are always associated with a poor prognosis. In the cases of bladder metastasis, disease progression was rapid after diagnosis. In the case of penile metastasis, the patient presents a longer overall survival than what was expected, according to the literature. Haematuria is a sign often associated with malignancy, primary or secondary, so in clinical practice, it should be correctly investigated.
